# The Deceptive Presentation of Myocardial Stunning: A Case of Suspected Stroke Preceding In-Hospital Cardiac Arrest

**DOI:** 10.7759/cureus.98168

**Published:** 2025-11-30

**Authors:** Mosaab A Okache, Hanouf I Abdelrahman, Ibtihal Mohamed, Fatima H Abbas, Reem A Mohmed

**Affiliations:** 1 Internal Medicine, Almana General Hospital, Dammam, SAU; 2 Emergency Medicine, Almana General Hospital, Dammam, SAU

**Keywords:** anticoagulation, cardioembolic stroke, hemorrhagic transformation, myocardial stunning, post-cardiac arrest care

## Abstract

Myocardial stunning is a reversible form of cardiac dysfunction that can follow cardiac arrest. We report a case where its presentation was masked by a primary neurological complaint. A 67-year-old male with cardiovascular risk factors presented to the emergency department with acute profound confusion and amnesia, initially suspected to be an acute ischemic stroke. Within one hour, his condition deteriorated, and he suffered an in-hospital cardiac arrest due to pulseless ventricular tachycardia, requiring prolonged resuscitation. Post-return of spontaneous circulation, comprehensive neuroimaging, including CT angiography and MRI, conclusively ruled out an acute cerebrovascular event. The working diagnosis was revised to a stroke mimic of undetermined etiology. Workup revealed severe left ventricular dysfunction consistent with global myocardial stunning. The patient's cardiac function and mental status gradually recovered with supportive care. This case highlights that acute, primary cardiac pathology can manifest with dramatic neurological symptoms, leading to initial misdiagnosis. It underscores the importance of considering myocardial stunning in any patient with neurological symptoms and cardiovascular instability, as rapid deterioration can occur.

## Introduction

The initial assessment of acute neurological deficits in the emergency department is a high-stakes endeavor. While acute ischemic stroke is a leading concern, a significant proportion of these presentations are later identified as stroke mimics, with reported frequencies ranging from 20% to 38% [1]. Common mimics include metabolic encephalopathy, seizures, and syncope. Differentiating a mimic from a true stroke is critical, as misdiagnosis can lead to inappropriate therapy and delayed treatment of the underlying condition [2].

A particularly perilous scenario occurs when the mimic is a prodrome to a catastrophic cardiovascular event. Myocardial stunning is a well-described phenomenon of transient, severe biventricular systolic dysfunction that follows an ischemic insult, such as cardiac arrest, even in the absence of acute coronary occlusion [3]. The initial clinical presentation of myocardial stunning is variable and can include non-specific symptoms like confusion, weakness, or dizziness, which can be easily mistaken for a primary neurological process [4].

We present a case of a patient whose presentation with acute confusion led to an initial suspicion of stroke, which was followed minutes later by a cardiac arrest. The subsequent workup revealed global myocardial stunning as the likely cause of both the arrest and the preceding neurological symptoms, with no evidence of an acute stroke. This case serves as a stark reminder of the diagnostic challenges at the intersection of neurology and cardiology.

## Case presentation

A 67-year-old male with a history of paroxysmal atrial fibrillation, hypertension, and diabetes mellitus presented to the emergency department with acute-onset profound confusion and amnesia. His home medications included Bisoprolol 5 mg daily, which had been discontinued five days prior, along with Metformin 500 mg twice daily and Apixaban 5 mg twice daily for stroke prophylaxis. Initial examination revealed a disoriented patient with amnesia but no focal neurological deficits, prompting initial concern for acute ischemic stroke.

Initial workup and clinical deterioration

**Figure 1:**
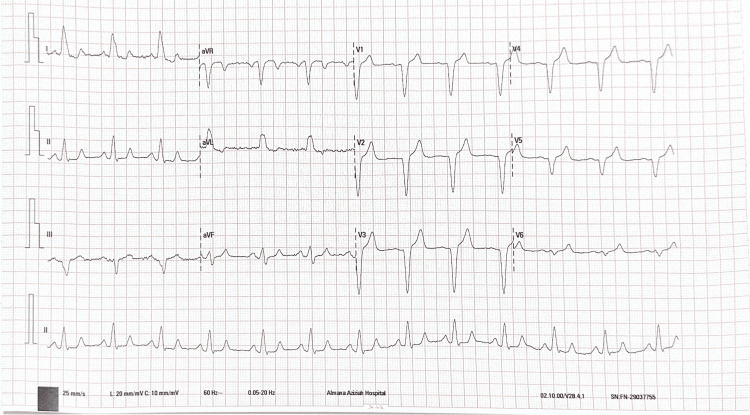
ECG showing atrial fibrillation with new left bundle branch block aVR: augmented vector right, aVL: augmented vector left, aVF: augmented vector foot.

The following diagnostic investigations were performed during the initial evaluation: non-contrast CT brain, no acute intracranial pathology, electrocardiogram (ECG), and atrial fibrillation with new left bundle branch block (Figure 1).

Blood investigations

Random blood sugar is 244 mg/dL; venous blood gas shows a pH of 7.37, partial pressure of carbon dioxide (pCO_2_) of 38.4 mmHg, and HCO_3_ of 21.9 mmol/L; and complete blood count reveals a WBC count of 12.5 × 10⁹/L, hemoglobin of 13.8 g/dL, and platelets of 280 × 10⁹/L. Approximately one hour after presentation, before any stroke-specific medications were administered, the patient's condition deteriorated acutely. He suffered cardiac arrest with initial rhythm of pulseless ventricular tachycardia. After 16 minutes of advanced cardiac life support, including defibrillation and adrenaline administration, return of spontaneous circulation was achieved.

Post-resuscitation management and investigations

**Figure 2:**
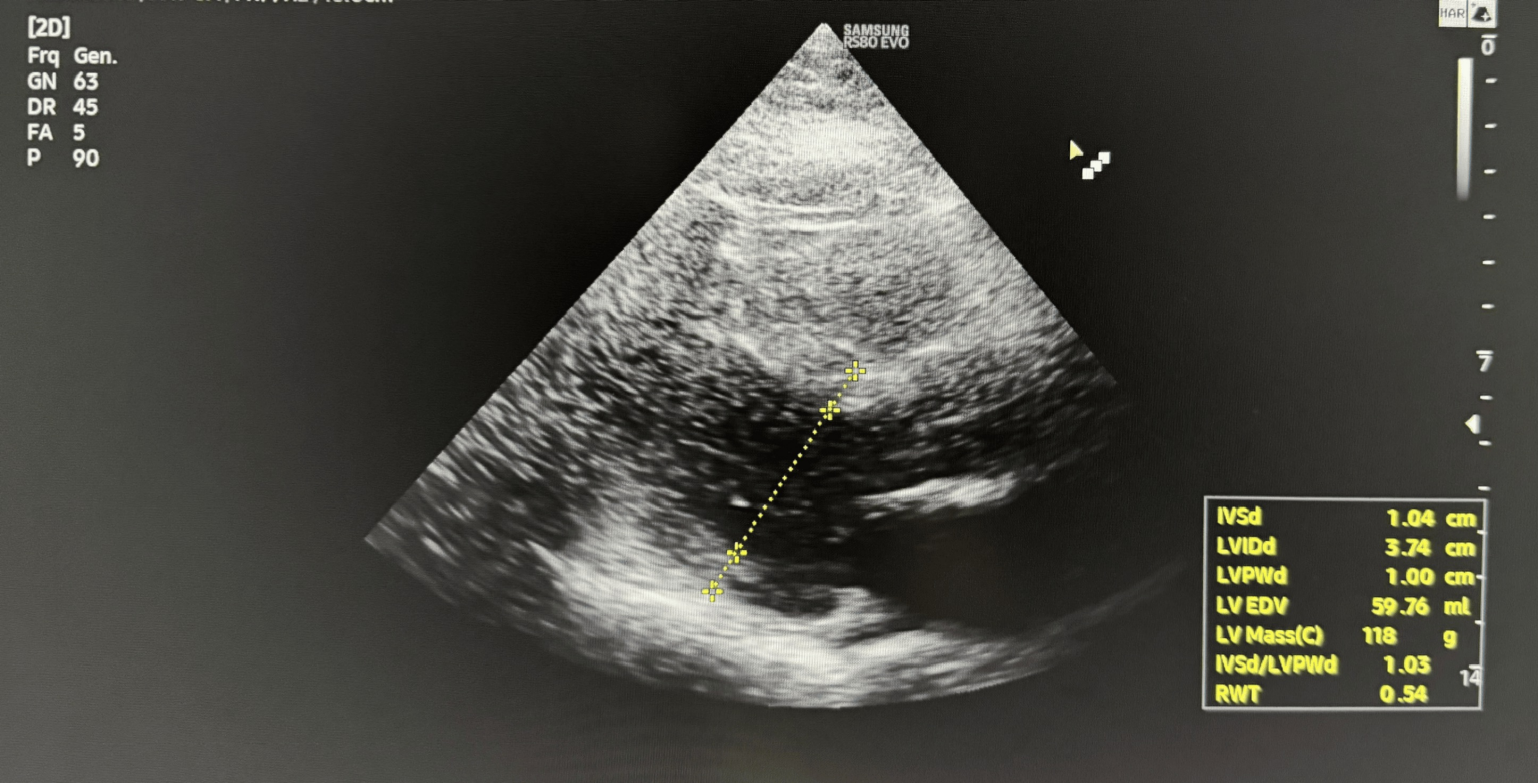
Echocardiogram showing severe global left ventricular dysfunction

The patient was transferred to the intensive care unit for post-cardiac arrest care. Additional investigations revealed: bedside echocardiogram demonstrated severe global left ventricular dysfunction (EF, 30%-35%) consistent with myocardial stunning (Figure 2).

Chest X-ray

Chest X-ray showed clear lung fields with appropriate endotracheal tube placement.

Coronary angiography

**Figure 3:**
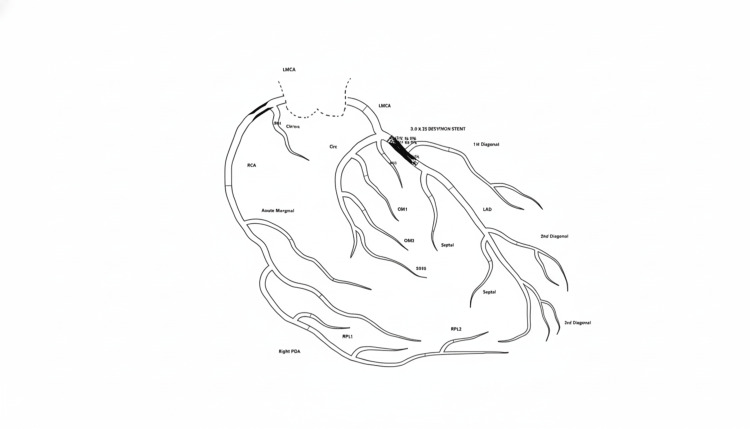
Coronary angiogram demonstrating critical LAD lesion Original schematic diagram created at Almana General Hospital. LAD: left anterior descending artery, LMCA: left main coronary artery, RCA: right coronary artery, OM: obtuse marginal artery, POA: posterolateral artery, RPL: right posterolateral artery.

Coronary angiography revealed a significant multi-vessel coronary artery disease with a critical proximal left anterior descending artery (LAD) lesion (Figure 3).

Hospital course and outcome

The multidisciplinary management included hemodynamic support to maintain mean arterial pressure (MAP) >65 mmHg; dual antiplatelet therapy, Aspirin 100 mg daily and Clopidogrel 75 mg daily; anticoagulation, Enoxaparin 40 mg subcutaneously daily for thromboembolic prophylaxis; seizure prophylaxis, Levetiracetam 500 mg intravenously twice daily; statin therapy, Atorvastatin 40 mg daily; glycemic control with insulin sliding scale; and the re-initiation of Bisoprolol 2.5 mg daily for heart rate control. The patient then underwent successful percutaneous coronary intervention with stenting to the LAD artery. His neurological status gradually improved, and follow-up echocardiogram showed significant recovery of left ventricular function (EF, 48%). He was discharged on Aspirin 100 mg daily, Clopidogrel 75 mg daily, Apixaban 2.5 mg twice daily, Bisoprolol 5 mg daily, and Atorvastatin 40 mg daily with planned outpatient follow-up.

## Discussion

This case provides a powerful illustration of how a primary cardiac event can masquerade as a primary neurological disorder, presenting a significant diagnostic challenge. The patient's acute confusion and amnesia, in the context of his risk factors, understandably triggered a stroke alert. This initial diagnostic suspicion is common, as stroke mimics constitute a substantial portion of acute neurological presentations, with studies reporting frequencies ranging from 20% to 38% [1]. Our case aligns with existing literature that identifies cardiovascular events as a critical category of stroke mimic, often presenting with non-specific neurological symptoms such as syncope or encephalopathy [2].

The rapid progression to cardiac arrest and the absence of acute findings on comprehensive neuroimaging forced a critical re-evaluation. The initial neurological symptoms were almost certainly a manifestation of global cerebral hypoperfusion due to the evolving severe left ventricular dysfunction from myocardial stunning. This pathophysiological mechanism is well-documented; myocardial stunning describes a transient, severe biventricular systolic dysfunction that follows an ischemic insult, such as cardiac arrest, even in the absence of an acute coronary occlusion [3]. The clinical presentation of this condition is variable and can include non-specific symptoms like confusion and weakness, which can be easily mistaken for a primary neurological process [4]. The cessation of his beta-blocker five days prior may have contributed to a catecholamine surge, exacerbating the strain on a vulnerable myocardium and precipitating the malignant arrhythmia, a factor often noted in cases of sudden cardiac deterioration following abrupt withdrawal of beta-blockade [5].

The key learning point from this case is the paramount importance of maintaining a broad differential diagnosis, even when the presentation seems classic for a single condition. While stroke protocols are essential for rapid treatment, this case highlights that the "time is brain" mantra must be balanced with "time is myocardium" in the right clinical context. The swift exclusion of a stroke via neuroimaging allowed the clinical team to pivot and focus on the true, life-threatening cardiac pathology. This approach is supported by guidelines that emphasize the need for simultaneous assessment of cardiac and neurological systems in patients presenting with altered mental status of uncertain etiology [6]. Myocardial stunning should be considered in the differential for any patient with acute neurological symptoms and cardiovascular instability, as its recognition is crucial for accurate prognostication and appropriate management, preventing the pitfalls of diagnostic anchoring.

## Conclusions

This report underscores several critical principles in emergency and critical care medicine. First, it reinforces that stroke mimics are common and can be the heralding sign of a severe systemic illness, including cardiac pathology. Second, it demonstrates the dramatic presentation of myocardial stunning, which can include profound neurological deficits preceding hemodynamic collapse. Finally, it emphasizes that initial diagnostic impressions must be continuously questioned and revised in the face of new data, particularly when the clinical course is rapidly progressive. A high index of suspicion for cardiac causes in apparent stroke presentations can be life-saving.
